# German S3 guideline on the use of dental ceramic implants

**DOI:** 10.1186/s40729-022-00445-z

**Published:** 2022-10-03

**Authors:** D. G. E. Thiem, D. Stephan, K. Kniha, R. J. Kohal, S. Röhling, B. C. Spies, M. Stimmelmayr, K. A. Grötz

**Affiliations:** 1 Department of Oral and Maxillofacial Surgery, Facial Plastic Surgery, University Medical Centre Mainz, Augustusplatz 2, 55131 Mainz, Germany; 2 Private Practice for Oral Surgery and Implantology, Rosental 6, 80331 Munich, Germany; 3 Department of Prosthetic Dentistry, University Medical Centre Freiburg, Hugstetter Straße 55, 79106 Freiburg, Germany; 4 Private Praxis for Oral Surgery, Oralchirurgie T1, im Schäfflerhaus Theaterstr. 1, 80333 Munich, Germany; 5 Private Practice for Oral Surgery, Josef-Heilingbrunner-Straße 2, 93413 Cham, Germany; 6 Helios Dr. Horst Schmidt Kliniken Wiesbaden, Ludwig-Erhard-Straße 100, 65199 Wiesbaden, Germany

**Keywords:** Zirconia dental implants, Zirconia, Dental implantology, German guideline, Evidence, Clinical evidence

## Abstract

**Purpose:**

Based on the excellent long-term data, dental implants made of titanium are considered the international implantological standard for replacing missing teeth. However, ceramic implants made of zirconia (ZrO_2_) have experienced a renaissance in the last 15 years due to constant innovations in materials and products, with material properties and soft tissue- and osseointegration behavior comparable to those of titanium. However, one limitation concerning ceramic implants is the lack of reliable long-term data, especially in the case of two-piece implant systems. As there is an increasing demand for ceramic implants from practitioners and patients, the German Society for Implantology (DGI) has decided to develop a guideline on the use of dental ceramic implants at the highest available evidence level with the involvement of experts in this field.

**Methods:**

Statements and recommendations were prepared after conducting a systematic literature search and an independent assessment process involving the relevant clinical literature from 2008 to 2021. The adopted recommendations and statements are summarized in this guideline.

**Results and conclusions:**

It confirms the feasible use of one-piece zirconia implants as an addendum/alternative to titanium implants. No final conclusion regarding the application of two-piece ceramic implant systems could be drawn on the basis of the existing data, thus its use can only be recommended after the patient has been informed in detail about the lack of long-term clinical data.

## Background

Dental implant treatment has been proven to be successful in oral rehabilitation with the usage of titanium-based implants which are regarded as gold standard. For the past decade, product innovations and material improvements have led to increased importance of implants based on zirconia which are now considered a therapeutical alternative to titanium implants more often. Due to an increased interest from both sides, from dentists as wells as patients, the *German Implantology Society* (DGI) together with the *German society for Dental and Oral Medicine* (DGZMK) has developed a new S3 guideline for the use of zirconia dental implants according to the current evidence.

## Methods

The process of developing, creating and updating guidelines is based on the currently applicable regulations of the *Association of the Scientific Medical Societies in Germany* (AWMF) which in turn refers to *The Appraisal of Guidelines for Research and Evaluation* (AGREE ll). In brief, a specific research question was designed according to the PICO scheme (*How can the use of ceramic implants be evaluated with regard to implant survival and implant success for the replacement of missing teeth at the present time?*). Subsequently to the selection of authors based on their scientific focus as wells as considering their personal conflict of interest, a systematic literature research was performed. In total, 8 prospective clinical studies, 2 reviews and 1 meta-review were identified in the period from January 01, 2018 to August 31, 2021. The publications were analyzed and critically evaluated with regard to the research question above. Finally, a structured consensus conference with all relevant dental and medical societies took place. The results of the vote are published as a guideline with practical treatment recommendations and statements as an aid to decision-making in everyday clinical practice. Furthermore, an internal quality management was applied in order to secure the high quality of the final guideline.

### Material properties and composition

**Fig. 1:**
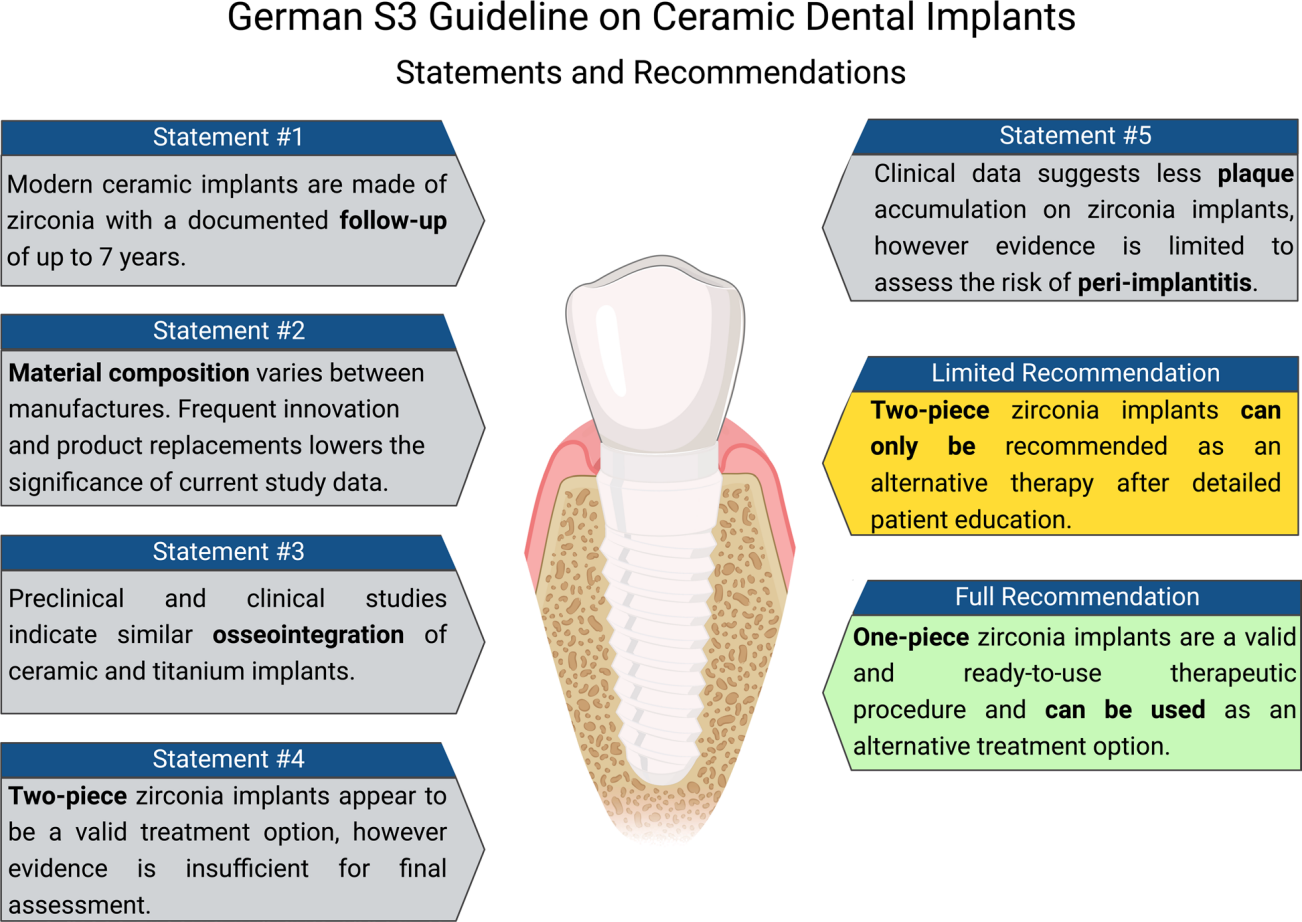
Illustration of the contents and recommendations of the S3 guideline on ceramic implants

The following article provides an overview on the new guideline for the use of zirconia dental implants. In general, two types of dental implants can be distinguished in terms of the used materials. In the past, besides titanium there were implants based on aluminum oxide [1] which, due to their increased fracture rate, have not found their way into clinical practice. However, in 2001 zirconium dioxide (=zirconia) was introduced and still represents the most frequently used base material for dental ceramic implants today. It is subject to continuous development of materials and production processes, though. On the one hand, constant innovation leads to increased implant quality and improved material properties with the aging process being only of secondary importance in clinical practice for example [2]. The material properties regarding bending capacity (900–1200 MPa) and fracture toughness (6–9 MPa) are enough for clinical application, whereas toughness is much higher in titanium implants [3]. On the other hand, constant material and thus product renewals have a negative influence on the study situation, as the assessment of long-term data beyond 5 years is thus made impossible [4–6]. Material composition of zirconia-based dental implants further appears to be depending on the manufacturer and the values of investigations are reduced by the continuous change of material compositions and product replacements [7–12]. Implant survival and the success of an oral rehabilitation is affected by numerous variables: the individual condition of each patient is one of them as well as possible peri-operative complications; e.g., biomechanical overload, resulting in loosening of the implant or implant fracture [13] as well as peri-implantitis [14] (Fig. 1).

### Osseointegration

Osseointegration is the prerequisite for implant success and is considered to be completed after an average of 8 to 12 weeks in terms of sufficient secondary stability [15, 16]. The dynamics of osseointegration can be influenced by the modification of the implant surface [17–20]. Zirconia-based dental implants with a microrough surface are known to not only reduce the time needed for bone formation, but also increase bone stability. Osseointegration of zirconia-based dental implants is therefore considered to be similar to titanium implants [4, 10, 12, 21–23] (Fig. 1).

### Plaque accumulation and peri-implantitis

Peri-implantitis is an inflammatory process around an osseointegrated implant that includes soft tissue inflammation and progressive loss of supporting bone beyond the state of biological bone remodeling. The accumulation of plaque usually precedes this clinical scenario. Plaque represents the prerequisite for inflammatory processes around the implant possibly resulting in peri-implantitis which is considered to be the most common cause late implant loss [24]. Although initial clinical evidence showed less plaque accumulation and thus a reduced risk of peri-implantitis with ceramic implants compared to titanium implants, the available clinical evidence is not yet sufficient to conclusively draw conclusions regarding this complex interaction [25, 26] (Fig. 1). The first clinical evidence of a lower risk of peri-implantitis with ceramic implants was obtained in a clinical prospective study of a patient population comparing ceramic and titanium implants. The highest bacterial load was found around titanium implants, followed by the zirconia implant and the natural tooth. At the same time, the peri-implant soft-tissue inflammation was highest around the examined titanium implants [26]. These results were confirmed in a recent randomized comparative clinical trial (RCT) in 42 patients also comparing ceramic and titanium implants [25].

### Recommendations for the therapeutical use of zirconia implant

Ultimately, treatment success depends on implant survival. Dental implants can either consist of one or two pieces. One-piece implants require transgingival healing and a maximum precision planning, since they offer compensation possibilities when the implant axis is not aligned perfectly. Clinical studies demonstrated high success rates (~ 97%) for one-piece zirconia-based dental implants over a follow-up period of more than 7 years. Hence, they can be recommended as therapeutical alternative for the replacement of missing teeth [4, 5, 23, 27–32]. In contrast, two-piece implants offer a better possibility of simultaneous bone augmentation and load-free healing due to submucosal positioning. In addition, two-piece systems offer more flexibility and a wider range of prosthetic restoration options. Commercially available two-piece zirconia-based ceramic implants can be recommended at this time as an alternative treatment option for replacing missing teeth either under study conditions and/or after appropriate detailed patient education. It is, however, not possible to finally assess their general suitability due to the missing scientific clinical evidence from long-term studies [11, 12] (Fig. 1).

## Conclusions

In summary, the data on the use of modern zirconia-based ceramic implants have significantly improved in recent years. However, constant improvements of material properties and related product updates negatively affected and affect the availability of reliable long-term data, particularly in the case of two-piece implants. Therefore, more long-term clinical studies are required to assess the use of dental ceramic implants more reliably.

## Data Availability

The datasets used and/or analyzed during the current study are available from the corresponding author on reasonable request.
