# Correction: German S3 guideline on the use of dental ceramic implants

**DOI:** 10.1186/s40729-022-00465-9

**Published:** 2023-01-17

**Authors:** D. G. E. Thiem, D. Stephan, K. Kniha, R. J. Kohal, S. Röhling, B. C. Spies, M. Stimmelmayr, K. A. Grötz

**Affiliations:** 1 Department of Oral and Maxillofacial Surgery, Facial Plastic Surgery, University Medical Centre Mainz, Augustusplatz 2, 55131 Mainz, Germany; 2 Private Practice for Oral Surgery and Implantology, Rosental 6, 80331 Munich, Germany; 3 Department of Prosthetic Dentistry, University Medical Centre Freiburg, Hugstetter Straße 55, 79106 Freiburg, Germany; 4 Private Praxis for Oral Surgery, Oralchirurgie T1, im Schäferhaus Theaterstr. 1, 80333 Munich, Germany; 5 Private Practice for Oral Surgery, Josef-Heilingbrunner-Straße 2, 93413 Cham, Germany; 6 Helios Dr. Horst Schmidt Kliniken Wiesbaden, Ludwig-Erhard-Straße 100, 65199 Wiesbaden, Germany

**Correction: International Journal of Implant Dentistry (2022) 8:43** https://doi.org/10.1186/s40729-022-00445-z

The original publication of this article [1] contained an incorrect author name. The incorrect and correct information is available in this correction article. The original article has been updated.

- **Incorrect:** C. B. Spies
- **Correct:** B. C. Spies
